# Designing for implementation: user-centered development and pilot testing of a behavioral economic-inspired electronic health record clinical decision support module

**DOI:** 10.1186/s40814-019-0403-z

**Published:** 2019-02-20

**Authors:** Sara Kuppin Chokshi, Hayley M. Belli, Andrea B. Troxel, Saul Blecker, Caroline Blaum, Paul Testa, Devin Mann

**Affiliations:** Department of Population Health, NYU School of Medicine, 227 E. 30th St., 7th Fl, New York, NY 10016 USA

**Keywords:** Electronic health records, Behavioral economics, Clinical decision support, Diabetes

## Abstract

**Background:**

Current guidelines recommend less aggressive target hemoglobin A1c (HbA1c) levels based on older age and lower life expectancy for older adults with diabetes. The effectiveness of electronic health record (EHR) clinical decision support (CDS) in promoting guideline adherence is undermined by alert fatigue and poor workflow integration. Integrating behavioral economics (BE) and CDS tools is a novel approach to improving adherence to guidelines while minimizing clinician burden.

**Methods:**

We will apply a systematic, user-centered design approach to incorporate BE “nudges” into a CDS module and will perform user testing in two “vanguard” sites. To accomplish this, we will conduct (1) semi-structured interviews with key informants (*n* = 8), (2) a 2-h, design-thinking workshop to derive and refine initial module ideas, and (3) semi-structured group interviews at each site with clinic leaders and clinicians to elicit feedback on three proposed nudge module components (navigator section, inbasket refill protocol, medication preference list). Detailed field notes will be summarized by module idea and usability theme for rapid iteration. Frequency of firing and user action taken will be assessed in the first month of implementation via EHR reporting to confirm that module components and related reporting are working as expected as well as assess utilization. To assess the utilization and feasibility of the new tools and generate estimates of clinician compliance with the Choosing Wisely guideline for diabetes management in older adults, a 6-month, single-arm pilot study of the BE-EHR module will be conducted in six outpatient primary care clinics.

**Discussion:**

We hypothesize that a low burden, user-centered approach to design will yield a BE-driven, CDS module with relatively high utilization by clinicians. The resulting module will establish a platform for exploring the ability of BE concepts embedded within the EHR to affect guideline adherence for other use cases.

**Electronic supplementary material:**

The online version of this article (10.1186/s40814-019-0403-z) contains supplementary material, which is available to authorized users.

## Background

Intensive glycemic control is of unclear benefit and carries increased risk for older adults (defined here as 76 years and older) with diabetes [1]. A number of randomized controlled trials, including the Action to Control Cardiovascular Risk in Diabetes (ACCORD) trial [1], the Action in Diabetes and Vascular Disease: Preterax and Diamicron Modified Release Controlled Evaluation (ADVANCE) trial [2], and the Veterans Affairs Diabetes Trial (VADT) [3], found that intensive glycemic control was not protective for macrovascular complications of diabetes including myocardial infarction or stroke. These trials demonstrate the potential for harm with tight glycemic control, notably increased risk of hypoglycemia [4], and a suggestion of increased all-cause mortality [1]. Older adults are particularly susceptible to harms related to hypoglycemia in diabetes, including emergent hospitalization and neurologic complications [5–9]. Intensive glycemic treatment may also lead to increased risk of polypharmacy and adverse medicine interactions for older adults with multiple chronic conditions [10].

### Choosing Wisely guideline for older adults with diabetes

Due to evidence that tight glycemic control in older adults with diabetes may be medically harmful, in 2013 (revised in 2015), the American Geriatrics Society (AGS) developed ten Choosing Wisely (CW) guidelines, of which the third guideline states the following: “Avoid using medications other than metformin to achieve hemoglobin HbA1c<7.5% in most older adults; moderate control is generally better.” [11] Specifically, this guideline incorporates the balance between the number of comorbidities and life expectancy of older adults to provide target ranges for glycemic goals. Reasonable targets include an HbA1c of 7.0–7.5% in healthy, older adults with long life expectancy; 7.5–8.0% for patients with moderate comorbidity and a life expectancy of less than 10 years; and 8.0–9.0% for patients with multiple comorbid conditions and a shorter life expectancy [4, 12].

These recommendations build on previous work categorizing older adults with diabetes into three clinical groups based on health status [13] and have been endorsed by numerous expert panels and guidelines [8, 14, 15]. The target ranges for glycemic control are similar to HbA1c values that have been associated with the best outcomes for older adults with comorbid conditions in observational or modeling studies [16–18]. The American Diabetes Association (ADA) and others have recommended similar health status categories related to HbA1c targets in older adults [8, 14, 15]. Although the ADA guidelines do not identify less aggressive targets for HbA1c [8], other societies, including AGS, recommend similar less aggressive thresholds for glycemic control [15, 19–21].

Despite the CW recommendations, a substantial number of older adults have intensive glycemic control that may not be necessary [22–24]. Additionally, older patients with intensive glycemic control generally do not undergo de-intensification of therapy, suggesting opportunity for improving appropriate care [25]. To combat this problem, the proposed study utilizes a unique approach: using behavioral economics via electronic health records to influence provider behavior with respect to diabetes care for older adults.

### Behavioral economics and the EHR

The field of behavioral economics (BE) combines principles from economics and psychology to recognize the limitations of the classical economic framework that views human decision-makers as purely rational actors [26]. In reality, humans are predictably irrational [27], making common decision errors that are explicable through a set of psychological principles, and are therefore *predictable*. Traits contributing to decision errors include loss aversion, anchoring, overweighting of small probabilities, present bias, regret aversion, sensitivity to defaults, and the power of social comparisons [28]. Once recognized, each of these decision errors can be harnessed and overcome, often in the form of gentle “nudges” that make a desired behavior more likely [29–32].

Meanwhile, electronic health records (EHRs) now dominate the landscape, influencing nearly every clinical decision, workflow, and order placed by health care providers. Clinical decision support (CDS) is the primary EHR tool for influencing clinical decision-making and promoting adherence to clinical guidelines. CDS is an effective tool for improving provider performance and patient outcomes [33, 34]. Moreover, best practices for maximizing CDS effectiveness have been identified [35, 36]. Successful CDS must deliver accurate information in the right clinical context at the point of care and must be integrated into the relevant provider’s workflow [37]. Large, systematic reviews of CDS have demonstrated a moderate ability to reduce morbidity, utilization, and costs [34, 38]. These modest improvements, however, are undermined by the well-documented problems of alert fatigue and poor workflow integration, which together blunt the potential of the EHR and CDS to improve healthcare outcomes [39].

Integrating behavioral economics strategies and electronic health records using various CDS tools is a novel approach to improving guideline adherence that also seeks to minimize negative impacts on clinical workflow and cognitive load. For example, Meeker et al. integrated three BE concepts (suggested alternatives, accountable justification, and peer comparisons) into the EHR at 50 primary care practices to significantly (~ 5–7%) reduce inappropriate antibiotic prescribing for upper respiratory infections [40]. New approaches like these are needed to complement the traditional alerts, reminders, and other CDS tools that disrupt clinical workflow, increase cognitive load, and stress the limited capacity of clinicians to rationally process and evaluate the diverse and competing demands on their attention. By leveraging the opportunity presented by the EHR to combine modalities and extend the power of BE, this project seeks to develop a scalable intervention to reduce overtreatment in older adults with diabetes with minimal negative impact on clinician workflow or cognitive workload.

The objective of this study is to develop and pilot test a scalable, EHR customization toolkit that applies behavioral economic insights to promote appropriate diabetes care in older adults based on the AGS’s CW guideline to reduce overtreatment for the benefit of older adults with diabetes.

## Methods/design

**Fig. 1:**
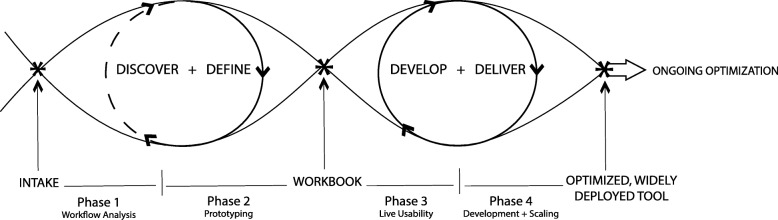
Process model for user-centered digital development

This study employs a pragmatic, user-centered approach as illustrated in Fig. 1 to achieve two aims: (1) develop new BE CDS modules to improve provider adherence to the CW guideline targeting overtreatment among older adults with diabetes and (2) test the effectiveness of these BE CDS modules in a naturalized, clinical setting.

### Aim 1: User-centered development of a behavioral economic-inspired CDS tool

Implementing a user-centered design process, we will incorporate behavioral economic approaches into existing CDS tools and clinical workflows to design, develop, iteratively refine, and user-test the module in two “vanguard” sites.

#### User-centered design of initial module (aim 1, phase 1)

The development and refinement process for the new BE CDS modules involves a series of linked steps that have been successfully used in previous CDS development studies [41–45]. The process includes user research, prototyping, and usability testing (see Fig. 1) [46, 47].

These processes will be used to identify or “discover” candidate BE concepts and potential CDS tools, which will then be analyzed according to their potential for workflow integration, their likely impact, and the complexity of their EHR development.

##### Identification of behavioral economic approaches and module opportunities and utilization measures

Initially, the BE and CDS literature will be further reviewed and candidate approaches selected for evaluation by the research team. Based on prior work and the current literature, Table 1 outlines example BE concepts and relevant CDS tools to support the implementation of each BE approach, along with utilization measures available to assess process outcomes.

**Table 1:** Behavioral economic strategies and associated module opportunity examples

BE strategy	Module opportunity	Utilization measure
Suggest alternatives [48]	Alert suggests metformin when trying to prescribe other diabetes medication in CW eligible patient	Percentage of eligible alerts where alternative is selected
Accountable justification [48]	Subcomponent of alert asking for justification if prescribing diabetes medication other than metformin	Percentage of eligible alerts where justification is provided (vs. only “clicked through”)
Defaults [30, 49]	Default all diabetes management order sets to suggest metformin in eligible patients	Percentage of order sets where clinician changes the default option
Anchoring [50]	Update HbA1c lab reports for CW eligible patients to show less aggressive treatment goals	Qualitative feedback and percentage of CW compliant patients pre- and post implementation update
Peer comparisons/norming [48, 51]	Modify clinician quality and safety dashboard to visualize diabetes management peer comparisons with color codes to indicate relative performance	Number of views of the peer comparison dashboard
Availability bias [30]	Medication preference list prioritizes metformin	Number of prescriptions for metformin initiated from preference list

##### Key informant interviews

The team will conduct key informant interviews with BE, CDS, and clinical experts using a semi-structured interview guide to explore (1) the potential impact of BE principles and strategies on improving guideline adherence for diabetes management in older adults, (2) the functionality of established CDS tools amenable to implementing the BE approach, (3) the clinical workflow footprint of the BE CDS nudges, and (4) how well candidate BE CDS nudges combine into a modular package of BE CDS tools.

##### Workflow analysis

The team will also conduct a workflow analysis adapted from the Agency for Healthcare Research and Quality (AHRQ) recommendations on workflow assessments [48]. We will conduct short observations in two selected vanguard clinic locations. The literature, interviews, and workflow assessments will be used in generation of the initial prototype BE-EHR module.

Workflows will be validated in a design-thinking workshop (described below), in follow-up interviews with key informants, and within-group feedback meetings with clinicians during user testing. During these follow-up meetings, any additional workflow variations that the module may need to support will be identified. Candidate workflows will then be discussed with the project team and other health system stakeholders to finalize the optimal workflow integration approach.

##### Design workshop

After initial key informant interviews and workflow analysis, a design-thinking workshop will be held to bring together findings from the interviews and workflow analysis. The design workshop will feature a structured, multidisciplinary workgroup composed of BE experts, CDS developers, relevant clinicians, and the research team. The sessions will be guided by a user-centered design facilitation protocol that sequentially leads the group through presentation and idea generation exercises around relevant behavioral economic concepts, CDS opportunities, and workflow obstacles and opportunities. Participants will be divided into small groups and provided materials and tasks designed to prompt new thinking on potential nudges. For example, the “crazy eights” exercise is an exercise in which individual team members are directed to draw eight ideas in 8 min. Each participant will be provided opportunities to “share back” their small group’s ideas as well as provide feedback on other groups’ ideas.

The primary goal of the workshop is to prompt divergent thinking or “discovery” (see Fig. 1) where participants focus on generating as many ideas as possible, as exemplified by the crazy eights exercise. Toward the end, the facilitator directs the group to “define” or converge on the best ideas for more explicit definition and development; to this end, the workshop will complete with the whole group voting on nudge ideas believed to be most promising. The session will be video- and audio-recorded, as well as summarized and converted into recommendations and revised workflow diagrams.

#### User testing (aim 1, phase 2)

Prior studies also established a clear link between the user-centered design process and successful implementation of clinical decision support tools within the EHR [49–51]. Upon completion of the design-thinking workshop and subsequent development of nudge prototypes, nudges will be further refined through a tailored, multi-phase feedback gathering and user testing process [46]. This pre-clinical testing serves as a clinical laboratory for building successful workflow-integrated tools with a high likelihood of adoption and adherence [41, 44, 52]. In addition to key informant interviews and group interviews with users as described above, a variety of methods for collecting both qualitative as well as quantitative user feedback will be selected. See Table 2 for user research methodologies to be employed on an as needed basis according to fit based on type of module, content, frequency of clinician “exposure,” and response to the module.

**Table 2:** Possible research methods for user testing

User testing methods	Description	Predicted frequency and time point
Key informant interviews	Semi-structured interviews with individual or small group (2–3) experts in primary care, geriatrics, EHR, diabetes, and other relevant fields	6–8 informants, 1–2 interviews each
Group interviews	Semi-structured interviews with groups of 4–6 clinicians	Pre- and post vanguard
“Ride along” observation	Individual observation session of clinician interaction with EHR real time, in situ	6–8 sessions per vanguard and pilot
Think-aloud usability testing [53]	Individual observations in “lab” of clinicians verbalizing all thoughts as they interact with the module following a carefully scripted series of EHR tasks	4–6 observations as needed in pilot
Near live [50, 53, 55]	Individual observations in “lab” of clinicians interacting with simulated data and patient actors to realistically model clinical use of module	4–6 observations as needed in pilot
Live [51, 56]	Individual observations of clinicians in situ using the tool in actual patient care	3 observations as needed in pilot

##### Development of algorithms to determine activation of CDS

In order to build a user-centered CDS tool that triggers appropriately for the target patient population, algorithms will be built-in to drive timing and content of module firings that incorporate both patient life expectancy (high, medium, low) and target glycemic index. A life expectancy algorithm (based on a scoring approach used by Quan et al. and prior research by DuGoff et al.) will be developed by the research team [53, 54]; it will be used to assign patients to low, medium, and high life expectancy categories based on patient characteristics including age, gender, and both the number and type of comorbidities. Depending on a patient’s life expectancy categorization, the individual will be assigned a target HbA1c range according to the specifications stated by the CW guideline targeting overtreatment of older patients with diabetes.

Developing an algorithm that accurately predicts life expectancy using electronic health record data poses challenges. One limitation is that the number and type of comorbidities recorded in the electronic medical record may be associated with the length of patient registration and frequency of visits. This would lead to under-reporting of comorbidities for some patients, which could lead to a higher predicted life expectancy categorization and consequential bias toward over-reporting compliance with the Choosing Wisely guidelines. While we are not able to validate the life expectancy algorithm by obtaining death certificate data, the peer-reviewed literature that we will utilize for building this algorithm is well-known and was created using data sources that verified accuracy using death certification data or sources directly applicable to analyzing EHR data. For example, Quan et al. developed a weighted scoring approach for the comorbidities using hospital and death certification data, and DuGoff et al. formulated their life expectancy tables assuming chronic conditions using Medicare beneficiary data from a sample of over 1.3 million patients [53, 54]. Therefore, we are confident that our adaptation of these algorithms will perform well in this setting.

Clinical decisions in accordance with the CW guideline will be drafted and approved by clinical experts on the research team and will guide actions prompted by text and visuals in the CDS modules. Example clinical actions include switching a patient’s medication to metformin, reducing the dose, or removing the prescription of an alternative medication for patients with an HbA1c laboratory value too tightly controlled given their calculated life expectancy. Once the new module has stabilized within the vanguard sites (workflow issues are resolved and clinician usage is stable) (aim 1), the tool will be activated at the pilot sites (aim 2).

### Aim 2: Pilot testing the BE-EHR modules (phase 3)

To assess the utilization and feasibility of the new tools and generate preliminary estimates of clinician compliance with the CW guideline, a 6-month, single-arm pilot study of the BE-EHR module will be conducted in six outpatient primary care clinics.

#### Intervention

The BE CDS module will trigger for appropriate patients according to the logic built into the module based on the patient’s age, life expectancy (as calculated by the algorithm described above), and current/target HbA1c. Based on these characteristics, the CDS may, for example, leverage the behavioral economic principle of defaults and suggest metformin if appropriate. Otherwise, the CDS will suggest not adding a new prescription if the HbA1c is above the lower bound of the target HbA1c threshold among the three life expectancy categories (7.0% for healthy patients, 7.5% for those with moderate comorbidity, and 8.0% for those with a shorter life expectancy [4, 12]). The CDS will suggest stopping or reducing the medication dose if the HbA1c is below the lower bound of the target HbA1c target threshold per life expectancy category, as that would indicate the patient’s HbA1c is being too tightly controlled. The BE CDS module (see Table 1) will be activated at the system level for all relevant diabetes EHR components. For example, a diabetes order set tailored to older adults might have default medications updated to be metformin and to reflect the CW-recommended glycemic targets for calculated life expectancy.

#### Setting and population

The New York University Langone Medical Center (NYULMC) primary care practices serve patients with a diverse range of socio-demographic characteristics; they range from faculty-based practices with predominately privately insured and/or Medicare patients to Federally Qualified Healthcare Centers with a large Medicaid population (see Table 3). For the pilot study, four practices will be recruited, purposely chosen to reflect key practice setting characteristics (number of full-time providers, depth of support staff, transition to medical home model, insurance mix, patient socio-demographics).

**Table 3:** Patient population

Primary care and endocrine practices (N)	78
Demographics	
% Black	15
% Hispanic	43
% Asian	9
% White	25
% Other	8
Patients ≥ 75 with diabetes	5187

Eligible patients within the practice sites will be those aged 76 or older with a diagnosis of diabetes. Patients will be categorized into one of the three glycemic target ranges inspired by the CW recommendation: (1) healthy older adults with an HbA1c target range of 7–7.5% and long life expectancy (defined here as 10+ years); (2) those with moderate comorbidity and a life expectancy of 3+ to 10 years, with a target range of 7.5–8%; and (3) those with multiple comorbidities and life expectancy of less than or equal to 3 years, with a target range of 8–9% [4]. These categories are determined using the life expectancy algorithm described earlier. We expect the majority of patients in the selected population to fall into the “high life expectancy” category (10+ years), as the average life expectancy of a 75-year-old is between 11 and 12 years [53], and about two thirds of patients in the Medicare population have multiple chronic conditions [54–57]. Twenty percent of the population is estimated to be moderately healthy older adults with a medium life expectancy, and 10% are estimated to have an end-stage illness, leading to low life expectancy.

#### Outcomes

Pilot study outcomes will measure implementation as well as provide initial estimates of the BE-EHR modules’ effectiveness.

Process outcomes include utilization, measured via the EHR, such as the percentage of alerts in which the clinician changes the default option. Other outcomes are noted in Table 1. We will also collect qualitative user feedback using user research methods deemed most appropriate (see Table 2) to understand clinicians’ experience with the BE-EHR module and evaluate the specific nudges, as well as explore acceptability, implementation, and adoption issues surrounding them, including assessing their impact on guideline-focused care for older adults with diabetes.

The primary clinical outcome is the percent of eligible patients who are compliant with the AGS CW guideline targeting overtreatment of older adults with diabetes. For each patient, the HbA1c level, diabetes medication prescription status, and life expectancy status will be determined. All patients whose HbA1c are within the target range for their life expectancy status will be considered compliant. Those whose HbA1c are below the target range and who are being treated with a non-metformin agent (i.e., are over-controlled) will be deemed non-compliant. If an eligible patient is on metformin only but the HbA1c is below the upper board of the target range, they will also be deemed non-compliant. These situations are delineated in Table 4. Patient-level compliance outcomes will be aggregated to produce a provider-level compliance rate as a proportion of each provider’s eligible patients.

**Table 4:** Choosing Wisely non-compliance categories

Measured HbA1c	Patient category	Current prescription
< 7	Healthy	Non-metformin agent
< 7	Moderate comorbidity	Non-metformin agent
< 7	Shorter life expectancy	Non-metformin agent
7–7.5	Moderate comorbidity	Non-metformin agent
7–7.5	Shorter life expectancy	Non-metformin agent
7.5–8	Shorter life expectancy	Non-metformin agent

### Statistical methods and analysis

#### Utilization rates

Utilization rates will be estimated for each provider for every BE module component to which they apply (see Table 1); these rates will be summarized across physicians with 95% confidence intervals. To assess clinical value, each patient will be classified as CW compliant or not using the definition above; this indicator will be averaged across patients within a provider to calculate a provider-level compliance rate. These will also be summarized with 95% confidence intervals. The pilot study will provide critical estimates of utilization rates and clinical outcomes, allowing for refinement of the clinical trial design (Additional file 1). Specifically, following completion of the pilot study, the pruning decisions described below can be made to eliminate less effective approaches and guide future module development decisions.

#### Pilot phase qualitative user feedback

As outlined in Table 2, user feedback collected in the pilot phase will be analyzed with both pragmatic and academic goals. In order to maintain the timeline necessary for iterative product development, all user feedback will be summarized from researcher notes, providing near real-time feedback for module development and iteration. Simultaneously, systematically collected user feedback will be analyzed for more detailed usability findings deemed valuable by the research team for module implementation and academic dissemination using Dedoose, a software package that can integrate transcripts, pictures, memos, and other materials. Procedures recommended by Patton and others that focus on developing coding protocols to highlight issues, problems, and potential recommendations will be used [58]. The goal of these analyses is to identify key barriers and facilitators, as well as any emerging themes related to the use of the BE-EHR module to influence provider adherence to the CW guidelines.

#### Intervention pruning

We will apply mixed methods “pruning criteria” to eliminate less effective approaches. To address the evaluation of individual components, utilization measures will be assessed to determine the value of the tool for incorporation into the eventual BE-EHR module. If the CDS tool is utilized in more than 30% of opportunities (e.g., if a suggested default is accepted in more than 30% of cases), then this tool will be retained in the module. While there is no gold standard threshold for what a “useful” CDS tool utilization rate should be, studies have noted common CDS tools with utilization rates between 5 and 40% depending on the type of CDS and the risk of ignoring it [59–61]. Furthermore, while low utilization rates may prove clinically worthwhile in high-risk patient safety situations, lower risk scenarios like the CW diabetes guidelines for older adults dictate the need for a stronger mean adoption rate [49]. Certain tools will not be evaluable with this criterion, for example, the effect of adjusting the language on HbA1c lab reports. For these tools, qualitative user feedback during usability testing and brief interviews with key informants during piloting will be used to complement the utilization pruning criterion.

#### Scalability and dissemination

The finalized BE-EHR module will guide other EHR users through a menu of customizable options that can switch on (or off) various BE-derived CDS tools to replicate the current intervention for improving clinician adherence to diabetes management guidelines in older adults. Additionally, to facilitate scalability and widespread dissemination, the tools will follow standards-based development approaches, enabling widespread adoption across healthcare systems and diverse EHR platforms. For information regarding other key components addressed in this study, please consult the Additional file 1: SPIRIT Checklist.

## Discussion

This study involves the development and pilot testing of an innovative CDS tool, implementing behavioral economic principles within the electronic health record to promote clinician adherence to the CW guideline for diabetes management in older adults. Interventions for diabetes care in older adults have typically focused on addressing under-treatment of hyperglycemia [62]; however, the intervention described presently focuses on a system-wide approach to reduce risks resulting from overtreatment of older adults with diabetes. Evidence and lessons learned from this study can potentially inform the design, testing, and implementation of similar interventions for other CW target conditions and beyond.

### Novelty of combining behavioral economics with clinical decision support tools

The incorporation of behavioral economic principles into EHR clinical decision support tools shows promise as a strategy to improve guideline adherence by addressing stubborn barriers, such as alert fatigue, that prevent the CDS from having a desired impact on clinician behavior. The proposed BE-EHR module will serve as a highly scalable platform for embedding a BE-based CDS into any EHR system. More importantly, this module can be easily applied to many other conditions in older adults and other populations where combining BE with EHR-based clinical decision support will be useful for improving guideline adherence such as AGS CW recommendations related to preventative screening procedures (e.g., colonoscopies), or increasing compliance to tobacco cessation.

Unlike most new CDS systems, we anticipate that the proposed BE-EHR module will have limited negative impact on clinical workflow and cognitive load. BE tools inherently bypass the central processing route that requires clinicians to actively think about decision-making [63]. Instead, these tools leverage the peripheral route, which uses contextual cues and other influencing tools to nudge clinicians to choose actions consistent with stated guidelines.

### Algorithm

While the present study aims to test the effectiveness of behavioral economic principles in the EHR as they relate to the overtreatment of elderly patients with diabetes, central to the CDS is the logic and clinical decision-making guided by the life expectancy algorithm and corresponding HbA1c targets. Such a clinical decision support tool currently does not exist within our vendor EHR (Epic™), suggesting a possible future area for development within the EHR. This life expectancy algorithm could easily be applied to other alternatives to clinical practice or additional CW guidelines as a tool to nudge clinician behavior across a variety of medical concerns affecting elderly adults.

### Important role of behavioral economics in achieving user-centered tools

Prior research, including that of the research team, show the value of taking a user-centered approach to development of decision support tools [41, 64, 65]. In doing so, the appropriate behavioral economic principles can be identified and strategies developed and deployed in a way that is most likely to leverage the motivations and workflows of end users (clinicians in the case of most CDS). Our multi-phased, mixed methods approach to design and user testing as well as multidisciplinary approach as supported by our diverse team and key informants (with expertise in behavioral economics, informatics, geriatrics, diabetes, social science, user research, and rigorous evaluation of clinical interventions) enables the design of a user-centered intervention more likely to achieve the adoption necessary to impact clinical outcomes.

### Limitations

As with any study design, there exist some limitations. First, the sample size that would be required to adequately power the present study is beyond the scope of this work. Hence, results from this pilot study will influence the decision of whether to push forward with a fully developed randomized controlled trial using only those nudges that show promise. Furthermore, with the incorporation of nudges (see Table 1) simultaneously across the EHR, it will be challenging to determine exactly which BE strategy or intervention has the strongest impact. Care will be taken to perform statistical analyses using adjustments and gathering summary statistics for different groups of nudges that are less likely to co-occur. Furthermore, we will collect information longitudinally to assess the order and timing with which clinicians were exposed to various nudges. As a pragmatic study, however, we are testing effectiveness in a real-world setting, for which clinicians in practice will be exposed to multiple nudges in combination or simultaneously, making the present design useful for assessing the overall impact in the EHR.

### Summary

In summary, the proposed research is a highly innovative, multi-phase study to determine the feasibility and impact of EHR-embedded BE approaches on provider adherence to the CW guideline for older adults with diabetes. The resulting BE-EHR module will establish a platform for exploring the ability of BE concepts embedded within the EHR to affect guideline adherence for other CW target areas. Moreover, it represents an exciting new channel for influencing provider behavior through less cognitively burdensome methods.

## Additional file

Additional file 1: SPIRIT Checklist. (DOC 123 kb)

## Abbreviations

ACCORD: Action to Control Cardiovascular Risk in Diabetes
ADA: American Diabetes Society
ADVANCE: Action in Diabetes and Vascular Disease: Preterax and Diamicron Modified Release Controlled Evaluation
AGS: American Geriatrics Society
AHRQ: Agency for Healthcare Research and Quality
BE: Behavioral economics
CDS: Clinical decision support
CW: Choosing Wisely
EHR: Electronic health record
HbA1C: Hemoglobin A1c
NYUMC: New York University Langone Medical Center
VADT: Veterans Affairs Diabetes Trial
